# Introduction to the special issue

**DOI:** 10.1186/s13722-020-0182-0

**Published:** 2020-02-05

**Authors:** Andrea Finlay, Ingrid Binswanger, Christine Timko

**Affiliations:** 1 Center for Innovation To Implementation (Ci2i), VA Palo Alto Health Care System, 795 Willow Road (MPD-152), Menlo Park, CA 94025 USA; 2 Department of Veterans Affairs, National Center on Homelessness Among Veterans, Menlo Park, CA USA; 3 Institute for Health Research, Kaiser Permanente Colorado, Denver, CO USA; 4 Colorado Permanente Medical Group, Denver, CO USA; 5 Division of General Internal Medicine, University of Colorado School of Medicine, Aurora, CO USA; 6 Department of Psychiatry and Behavioral Sciences, Stanford University School of Medicine, Stanford, CA USA

**Keywords:** Substance use disorders, Criminal justice, Treatments

## Abstract

This special issue of Addiction Science & Clinical Practice, “Addiction treatment access and utilization among criminal justice involved populations”, presents a series of articles on substance use disorder treatment access and utilization by people who have contact with the criminal justice system (e.g., jails, prisons, and courts). Despite the high prevalence of substance use disorders among people who experience these settings, evidence-based treatment for substance use disorders may be unavailable and/or care may be fragmented during transitions between settings. Articles in this special issue address several gaps in the literature and present a conceptual model of opioid overdose risk, the results of a randomized controlled trial to increase treatment uptake and retention during and after incarceration, descriptions of barriers to treatment after release from incarceration, and data from nationally representative surveys of substance use disorders and treatment use among people who have been involved in the criminal justice system. Importantly, the voices of people with lived experience in the criminal justice system were incorporated in two manuscripts. Together these articles advance our understanding of how to improve care coordination and expansion of services across systems and organizations to prevent overdose, improve treatment utilization, and ultimately, improve health outcomes among criminal justice involved populations in the United States who have substance use disorders or use substances.

## Background

Adults in the United States (US) in jails, prisons, or courts have a high prevalence of substance use disorders [1, 2] and are at risk for poor health outcomes related to substance use, such as opioid overdose [3]. Involvement in the criminal justice system (perhaps more accurately called the criminal legal system) occurs in many contexts, including interaction with law enforcement during arrest, participation in diversion programs or courts, incarceration in jails or prisons, and correctional supervision in the community. Transitions between criminal justice settings and the community can fragment substance use disorder care and lead to poor outcomes. The purpose of this special issue is to advance understanding of how to improve health outcomes among criminal justice populations who have substance use disorders or use substances in the US and international settings.

An estimated 58% of adults in US prisons and 63% in US jails have a substance use disorder, and 40% were using drugs at the time of committing the offense for which they were incarcerated [2]. Despite these statistics, and potential availability of evidence-based treatments, some substance use disorder treatments, such as pharmacotherapy, are difficult to access in criminal justice settings [4, 5]. In addition, engagement in treatment may decline once formerly justice involved people are no longer mandated to attend treatment [6].

## Special issue

This special issue of *Addiction Science & Clinical Practice*, “Addiction treatment access and utilization among criminal justice involved populations”, includes 10 articles that address substance use disorder treatment across criminal justice contexts, including prison, jails, and courts. We aimed to fill eight knowledge gaps among criminal justice involved populations: (1) Models for providing effective substance use disorder treatment and harm reduction; (2) Treatment and care coordination during the transition from incarceration to community settings; (3) The impact of criminal justice laws on substance use behavior, substance-related outcomes, and access to treatment and other services; (4) Overdose prevention; (5) Mental health and medical co-morbidities and their impact on substance use disorder treatment; (6) Health disparities in access to and utilization of substance use disorder treatment; (7) Patient-centered or technologically supported interventions to improve access to and utilization of effective substance use disorder treatment; and (8) Implementation approaches to increase uptake of evidence-based prevention and treatment practices. While more work is needed, the manuscripts in this special issue begin to inform some knowledge gaps, including models for providing effective substance use disorder treatment and harm reduction, treatment and care coordination during the transition from incarceration to the community, overdose prevention, and health disparities in access to and utilization of substance use disorder treatment.

Joudrey and colleagues contributed a novel conceptual model—The Post-Release Opioid-Related Overdose Risk Model—to guide an understanding of opioid-related overdose mortality after jail or prison release [7]. The model’s importance is that post-release opioid-related overdose mortality is the leading cause of death among people leaving jails or prisons. The model identifies underlying (e.g., chronic pain, HIV, trauma), intermediate (e.g., disrupted social networks, poverty, stigma), and proximate (e.g., opioid use, interrupted treatment, insufficient naloxone access) determinants of overdose mortality. Biological outcomes in the model include tolerance and overdose as well as mortality. One of the model’s implications is that mitigating overdose mortality risk requires improved coordination, tailoring, and expansion of services across systems and organizations.

Several articles in the special issue support aspects of Joudrey et al.’s conceptual model. They describe intervention programs and support services for patients with substance use disorders who were incarcerated or recently exited incarceration, including specific groups such as women. Two randomized controlled trials in the special issue focus on people incarcerated in prison. These studies aim to improve treatment initiation during incarceration and increase treatment continuation after release to the community. Ramsey et al. [8] present a protocol of a pilot study to reduce the risk of HIV infection among incarcerated women. The pilot was designed to increase uptake of pre-exposure prophylaxis (PrEP) during incarceration and linkage to community-based PrEP treatment upon release. Preventing HIV seroconversion is important because of the association between HIV positivity and risk of drug overdose [9]. Blue et al. [10] conducted secondary analyses to examine HIV risk behaviors among people incarcerated in prison who were randomized to receive buprenorphine in prison or in the community after release. Results indicate that participants who were randomized to receive buprenorphine in the community had a greater decrease in injection drug use than participants who were randomized to receive buprenorphine in prison. This study underscores the importance of ensuring immediate access to and utilization of substance use disorder treatment services once a person exits incarceration to reduce overdose.

Two studies on the Transitions Clinic Network, comprised of 19 medical clinics scattered across the United States that treat formerly incarcerated individuals, provide a model to support healthcare access and utilization among people exiting prison. Chamberlain et al. [11] applied a quantitative approach to identify factors associated with substance use soon after release from incarceration and suggested targeting interventions toward individuals with the greatest risk. Thomas et al. [12] conducted qualitative interviews with women who exited prison and attended a Transitions Clinic to examine how the clinic supported their treatment needs. The clinic improved women’s self-efficacy navigating healthcare systems and organizations as they re-enter the community. Together, these articles suggest that building partnerships between correctional systems and community healthcare organizations may assure smoother transitions for women and men being released from incarceration and reduce risks for overdose and other poor health outcomes.

Mixed methods and qualitative studies identified barriers to substance use disorder treatment access and utilization, which can inform intervention program design to address specific treatment needs of incarcerated populations. Using surveys of people exiting jails, Owens et al. [13] quantified barriers that contributed to challenges accessing substance use disorder treatment after release from jail. Important patient-level barriers included privacy concerns about speaking in a group, and system-level barriers included treatment waitlists. Using the social ecological model to guide analyses, Bunting et al. [14] interviewed social workers from the Kentucky Department of Corrections to identify patient-level barriers, such as lack of motivation, and system-level barriers, such as high case load and limited treatment resources. Implementation efforts to improve uptake of evidence-based substance use disorder treatment will need to tackle these barriers to ensure criminal justice involved populations can access substance use disorder care when desired.

Finally, three large nationally representative studies examined substance use and treatment among people with an incarceration history. Winkelman et al. [15] used National Survey on Drug Use and Health data to document a higher prevalence of tobacco use among individuals with a history of criminal justice involvement compared to those with no criminal history. Tobacco use remains the leading cause of preventable disease and death in the United States [16]. Winkelman et al.’s study should prompt the development and evaluation of new programs and policies to reduce tobacco-associated morbidity and mortality among criminal justice involved populations. Using data from the National Epidemiologic Survey on Alcohol and Related Conditions, Tsai and Gu [17] demonstrated that people with both a substance use disorder and an incarceration history had higher odds of utilizing substance use disorder treatment compared to people who had a substance use disorder without a history of incarceration. Consistent with Tsai and Gu’s results are the findings of Taylor et al.’s [18] national study of women with alcohol use disorder who used the Veterans Health Administration for care. Receipt of pharmacotherapy for alcohol use disorder was more frequent among women veterans with recent criminal justice involvement compared to women veterans with no known justice involvement. Together, these nationally representative studies inform our understanding of the treatment needs for criminal justice involved populations by providing some prevalence data on substance use disorders and treatment utilization.

## Research gaps

While the array of articles in this special issue begin to address some important gaps in the literature, many gaps remain. Patient perspectives on substance use disorder treatment access and utilization is relatively understudied [19]. Two articles in this issue—Thomas et al. [12] and Owens et al. [13] gave people with lived experience in the criminal justice system an opportunity to voice the challenges they faced when accessing substance use disorder treatment. However, more work is needed to address the life course and lived experience of criminal justice involved populations and how experiences influence both receipt and effectiveness of addiction treatment. Future care models designed to address substance use disorders in criminal justice populations should attend to these experiences to maximize the effectiveness of substance use disorder treatment.

There is a dearth of literature on criminal justice systems and substance use disorder treatment by country and criminal justice settings. Substance use treatment for criminal justice populations can vary widely by country, as well as within countries, and there may be existing international models that could inform the US research and treatment communities. The research in this issue primarily focuses on formerly incarcerated populations, but treatment for people on probation, parole, in jail, and in prison is also important. Studies that examine different treatments that are delivered (or not) in incarceration settings will inform the provision of care to correctional populations. Studies of other criminal justice contexts, such as law enforcement interactions and court systems, are also needed. Although there have been studies of drug courts [20], the delivery of substance use disorder treatment to individuals involved with other specialty courts, such as Veterans Courts, is unknown. Some law enforcement programs support diversion from the criminal justice system to treatment [21], the lessons learned from these programs could be disseminated broadly.

To address these gaps, the methodology used in studies on substance use disorder treatment of criminal justice populations could be improved. For example, definitions of criminal justice involvement vary across studies such that work to standardize a definition is needed. Broadly, the use and elaboration of conceptual models, such as the one initiated by Joudrey et al. [7] are needed to guide quantitative and qualitative research on substance use and its treatment among people in the criminal justice system. In addition to health services or treatment models from the medical literature, such as the Behavioral Model for Vulnerable Populations [22], conceptual or theoretical models from criminology could be applied to public health and medicine, such as the Sequential Intercept Model. This model was created to address the nexus of the criminal justice and mental health treatment systems [23]. Subsequent research should draw on these frameworks to develop conceptual models that guide research on criminal justice involved populations to inform substance use treatment theory, practice, and policy.

## Conclusions

The prevalence of substance use disorders is high among people with a criminal justice history [2], but evidence-based treatments for substance use disorders are often unavailable or fragmented for the population. Relationships between criminal justice and community agencies should be strengthened to ensure substance use disorder treatment is available immediately following exit from incarceration and responsive to individuals’ lived experience. Finally, although patients with a criminal justice history engaged in more substance use disorder treatment than their non-involved counterparts [17, 18], the high prevalence of substance use disorders suggests that public health programming targeted at criminal justice involved populations is needed. The articles in this special issue addressed many gaps in the literature, including integrating the voices of people with lived experience, but important research gaps remain to address their needs.

## Data Availability

Not applicable.

## Abbreviations

PrEP: Pre-exposure prophylaxis
US: United States

## References

[1] Finlay AK, Smelson D, Sawh L, McGuire J, Rosenthal J, Blue-Howells J (2016). U.S. Department of Veterans Affairs Veterans Justice Outreach program: connecting justice-involved veterans with mental health and substance use disorder treatment. Crim Justice Policy Rev.

[2] Bronson J, Stroop J, Zimmer S, Berzofsky M (2017). Drug use, dependence and abuse among state prisoners and jail inmates, 2007–2009 (NCJ 250546).

[3] Binswanger IA, Blatchford PJ, Mueller SR, Stern MF (2013). Mortality after prison release: opioid overdose and other causes of death, risk factors, and time trends from 1999 to 2009. Ann Intern Med.

[4] Nunn A, Zaller N, Dickman S, Trimbur C, Nijhawan A, Rich JD (2009). Methadone and buprenorphine prescribing and referral practices in US prison systems: results from a nationwide survey. Drug Alcohol Depend.

[5] Matusow H, Dickman SL, Rich JD, Fong C, Dumont DM, Hardin C (2013). Medication assisted treatment in US drug courts: results from a nationwide survey of availability, barriers and attitudes. J Subst Abuse Treat.

[6] Perron BE, Bright CL (2008). The influence of legal coercion on dropout from substance abuse treatment: results from a national survey. Drug Alcohol Depend.

[7] Joudrey PJ, Khan MR, Wang EA, Scheidell JD, Edelman EJ, McInnes DK (2019). A conceptual model for understanding post-release opioid-related overdose risk. Addict Sci Clin Pract.

[8] Ramsey SE, Ames EG, Brinkley-Rubinstein L, Teitelman AM, Clarke J, Kaplan C (2019). Linking women experiencing incarceration to community-based HIV pre-exposure prophylaxis care: protocol of a pilot trial. Addict Sci Clin Pract.

[9] Green TC, McGowan SK, Yokell MA, Pouget ER, Rich JD (2012). HIV infection and risk of overdose: a systematic review and meta-analysis. AIDS.

[10] Blue TR, Gordon MS, Schwartz RP, Couvillion K, Vocci FJ, Fitzgerald TT (2019). Longitudinal analysis of HIV-risk behaviors of participants in a randomized trial of prison-initiated buprenorphine. Addict Sci Clin Pract.

[11] Chamberlain A, Nyamu S, Aminawung J, Wang EA, Shavit S, Fox AD (2019). Illicit substance use after release from prison among formerly incarcerated primary care patients: a cross-sectional study. Addict Sci Clin Pract.

[12] Thomas K, Wilson JL, Bedell P, Morse DS (2019). "They didn't give up on me": a women's transitions clinic from the perspective of re-entering women. Addict Sci Clin Pract.

[13] Owens MD, Chen JA, Simpson TL, Timko C, Williams EC (2018). Barriers to addiction treatment among formerly incarcerated adults with substance use disorders. Addict Sci Clin Pract.

[14] Bunting AM, Oser CB, Staton M, Eddens KS, Knudsen H (2018). Clinician identified barriers to treatment for individuals in Appalachia with opioid use disorder following release from prison: a social ecological approach. Addict Sci Clin Pract.

[15] Winkelman TNA, Vickery KD, Busch AM (2019). Tobacco use among non-elderly adults with and without criminal justice involvement in the past year: United States, 2008–2016. Addict Sci Clin Pract.

[16] Jamal A, King BA, Neff LJ, Whitmill J, Babb SD, Graffunder CM (2016). Current cigarette smoking among adults—United States, 2005–2015. MMWR Morb Mortal Wkly Rep.

[17] Tsai J, Gu X (2019). Utilization of addiction treatment among U.S. adults with history of incarceration and substance use disorders. Addict Sci Clin Pract..

[18] Taylor E, Timko C, Harris AHS, Yu M, Finlay AK (2019). Receipt of pharmacotherapy for alcohol use disorder by justice-involved women in the Veterans Health Administration. Addict Sci Clin Pract.

[19] Oliva EM, Maisel NC, Gordon AJ, Harris AH (2011). Barriers to use of pharmacotherapy for addiction disorders and how to overcome them. Curr Psychiatry Rep.

[20] Marlowe DB, Hardin CD, Fox CL (2016). Painting the current picture: a national report on drug courts and other problem-solving courts.

[21] Jarvis SV, Kincaid L, Weltge AF, Lee M, Basinger SF (2019). Public intoxication: sobering centers as an alternative to incarceration, Houston, 2010–2017. Am J Public Health.

[22] Gelberg L, Andersen RM, Leake BD (2000). The behavioral model for vulnerable populations: application to medical care use and outcomes for homeless people. Health Serv Res.

[23] Munetz MR, Griffin PA (2006). Use of the sequential intercept model as an approach to decriminalization of people with serious mental illness. Psychiatr Serv.

